# Preliminary perspectives on gene therapy in fragile X syndrome: a caregiver view

**DOI:** 10.1186/s11689-025-09629-1

**Published:** 2025-09-01

**Authors:** Sarah E. A. Eley, Sydni Weissgold, Andrew C. Stanfield

**Affiliations:** 1 University of Edinburgh, Patrick Wild Centre, George Square, Edinburgh, UK; 2 University of Edinburgh, Patrick Wild Centre and Simons Initiative for the Developing Brain, George Square, Edinburgh, UK

**Keywords:** Gene therapy, Fragile X syndrome, Qualitative, Questionnaire, Views, Treatment

## Abstract

**Background:**

There have been increasing numbers of clinical trials of medications for fragile X syndrome (FXS) in recent years, many targeted at proposed underlying cellular or circuit based mechanisms. As yet none of these have led to widespread changes in clinical practice. Genetic therapies represent a different therapeutic approach, which aim to address the genetic mechanisms by which FXS arises. Although not yet moving into human studies in FXS, this is an area of increasing research importance in neurodevelopmental conditions more broadly. It is important that families affected by FXS get the chance to give their views about future genetic therapies, given the potential controversies around genetic therapies.

**Methods:**

We developed a questionnaire to capture caregiver views around gene therapy in FXS. The questionnaire was developed alongside a group of parents / caregivers of a child with FXS to ensure the language used was appropriate and that it would allow a variety of views to be captured. The questionnaire contained questions around current knowledge of gene therapy, what families think of gene therapy and their views on gene therapy trials taking place. Responses were analysed by thematic analysis carried out by two of the authors with data from the questionnaires being grouped into themes and subthemes.

**Results:**

The questionnaire was completed by 195 individuals who are parents of, or who care for, someone with FXS. Respondents were primarily from the UK (60.5%) and the Americas (22.1%). The majority of dependants were male (86%).

Responses showed a strong interest from the Fragile X community in gene therapy trials taking place, with themes emerging around quality of life, outcomes and feelings. Hope for positive change was balanced against caution about unintended consequences, the newness of the treatment and tolerability.

**Conclusion:**

Overall, caregivers felt hopeful, excited and interested in the prospect of gene therapy potentially providing a new treatment option, but there was some trepidation about the potential effects. Taking caregiver views into account will help inform decisions around the development and testing of any future genetic interventions.

**Supplementary Information:**

The online version contains supplementary material available at 10.1186/s11689-025-09629-1.

## Background

Fragile X syndrome (FXS) is the most common inherited form of intellectual disability (ID) with a prevalence of between 1.4 and 4.1 per 10,000 males and 0.9 and 1.46 per 10,000 females [15]. As well as distinct physical features [4, 13], individuals with FXS commonly present with behavioural features including hyperarousal, impulsivity, self-injurious and aggressive behaviour, social avoidance and repetitive behaviours [4, 8, 9].

At present, the pharmacological treatments used in FXS are symptomatic in nature, meaning that, although they may help with certain difficulties, they do not address the underlying cause of the condition [20]. Recent years have seen increasing numbers of clinical trials of more targeted treatments for FXS, designed to address the underlying pathophysiology of the condition, although as yet none have progressed to licensed treatments [11]. An alternative targeted approach also under investigation, though not yet in clinical trials, is gene therapy– a medical technique which aims to treat conditions by altering genetic material.

Although there are no gene therapy trials currently ongoing in FXS in humans, early phase trials are ongoing in other genetic neurodevelopmental conditions, most notably Rett syndrome [16] and Angelman syndrome, the latter currently recruiting for a phase 3 trial (https://clinicaltrials.gov/study/NCT06617429). Laboratory studies in FXS models have also shown early positive results, although it remains unclear whether these findings will translate to humans. Studies in *Fmr1*-knockout mice show partial rescue of locomotive activity in males and improved social dominance in females using an adeno– associated virus (AAV) based gene therapy [22]. EEG results also show correction of abnormal slow wave activity in *Fmr1*-knockout mice, supporting the use of AAV based gene therapy as a treatment for FXS [10]. Much work remains to be done, however these results raise the possibility that gene therapy for FXS may become a reality in the foreseeable future.

Although holding potential for future benefit, gene therapy raises questions around safety, ethics, side effects and long term risks/outcomes [14]. It is therefore important that the views of affected communities are sought at an early point in the development of such interventions. A recent study reviewing family and caregiver perspectives on gene therapy in Rett syndrome [17] discussed a strong hope that gene therapy would provide a cure, as well as lead to improvement in language and motor skills. Caregivers expressed their willingness to accept risks associated with gene therapy. It is not clear however that these findings will be the case for other genetic conditions, especially those such as FXS where there is a very wide range of clinical presentations and lower levels of co-occurring physical conditions than are found in many neurodevelopmental disorders.

We therefore set out to collect family and caregiver views and attitudes towards gene therapy for the treatment of FXS using a questionnaire based qualitative approach. The information from this study can potentially be used to guide research and influence clinical trial development.

## Methods

A thirteen item qualitative questionnaire to be completed by caregivers of people with FXS was developed by the authors (SEAE & AS). A group of parents/caregivers of children with FXS were consulted prior to, and during, the development of the questionnaire to ensure that a range of viewpoints would be captured and that the language used was appropriate. The group consulted was made up of 10 individuals: 5 males and 5 females with a mix of age of dependents with FXS from 4 years old through to adulthood. The group were broadly positive about the topic area but suggested a move away from quantitative questions and the use of more free text boxes to capture the richness of views within the community. The questionnaire contained questions around current knowledge of gene therapy, what families think of gene therapy and their views on gene therapy trials taking place. It also asked questions about the person they care for, including behaviours that can be associated with FXS, and whether or not they had previously taken part in a clinical trial (Supplementary Material).

The study was reviewed by the University of Edinburgh medical school research ethics committee (EMREC) with ethical approval granted on 25th April 2024. Study participation was voluntary and confidential. Data collection occurred between May and July 2024.

### Recruitment

The questionnaire was held electronically on a secure platform, JISC online surveys, endorsed by the University of Edinburgh.

Information about participating in the research was circulated to people who care for someone with FXS through our research centre (The Patrick Wild Centre) mailing lists and via FXS-related charitable organisations. These included the UK Fragile X Society, a family support organisation in the UK, and FRAXA research foundation, a US based organisation which funds research into the development of treatments for fragile X. It was also posted on The Patrick Wild Centre social media and on related websites.

### Data analysis

The data were analysed using an inductive thematic analysis approach as outlined by Braun and Clarke (2006), and the process was facilitated using NVivo software (NVivo, 2020). The analysis was led by SEAE, with support from a second researcher, SW—both trained researchers with experience of thematic analysis.

Initially a semantic analysis was conducted and codes developed (Goddard, 2011). SEAE conducted an initial line-by-line reading of each response to generate open codes. These codes were developed inductively, without applying a pre-existing coding frame, allowing patterns to emerge directly from the data. These initial codes were then reviewed, grouped, and refined into themes and subthemes. A coding log was maintained throughout the process, documenting decisions and changes in code definitions to support transparency and traceability.

To enhance the reliability of the analysis, SW independently coded the responses inductively and then reviewed, grouped, and refined into themes and subthemes. The two researchers then met to compare coding, discuss discrepancies, and refine the definitions of emerging themes and subthemes. Discrepancies were resolved through collaborative discussion, guided by close reference to the original data and the overarching research question. In cases of disagreement, the original data were revisited together, and decisions were made based on consensus to ensure consistency in interpretation.

Following this, the full dataset was re-examined using the refined coding framework to assess how well the final themes and subthemes captured the breadth and depth of the data. Final themes were determined after evaluating their relevance across the entire dataset and ensuring they reflected the caregivers’ perspectives.

## Results

The questionnaire was completed by 195 parents and caregivers of someone with FXS. 60.5% of responses were from the UK, 8.2% from the rest of Europe, 22.1% were from the Americas, 3.1% from Australia/NZ and 2.1% from rest of world. The majority of dependants were male (86%). 21% of respondents’ dependants had previously been involved in a clinical trial.

When asked about initial reactions to gene therapy, words such as interested, excited and hopeful were used very often, with nervousness and trepidation being used by a few; one response used the word sad. Respondents were asked if they think research into gene therapy for FXS should occur and the majority of responses said yes (94.5%). When asked if respondents would consider gene therapy as a treatment option for the person they care for with a majority of people saying yes (66.7%) and words such as ‘definitely’ and ‘absolutely’ being used often. Some responses used words such as ‘possibly’ or ‘depends’ (23.6%) while only a few responses said no (10.1%). Notably, there was a very mixed understanding amongst responses about what gene therapy is and what it could potentially entail; although some caregivers described themselves as knowing a lot about it, some felt they had little knowledge of what would be involved or possible outcomes that might be expected.

Data from the questionnaires were grouped into themes and subthemes. The three themes that emerged from the data were: quality of life, outcomes and feelings. The themes and subthemes identified following coding are shown in Table 1. Descriptions of what was coded in each theme and subtheme is shown in Table 1.

**Table 1:** Description of themes

**Theme**	**Sub-theme**	Illustrative words/phrases used*
Quality of life	Future	“improve life”, “normal life”, “make life easier”, “too old for gene therapy to work?”, “change in personality”
	Symptoms	“improve cognitive function”, “alleviate symptoms”, “avoid difficulties”, “how would they adapt to change in themselves?"
	Tolerability	“method of delivery”, “tolerability of procedures”, “anxiety of child”, “pain”, “newness”
Outcomes	Cure	“address root cause”, “better future”, “reduce caregiver burden”, “help”, “fix”, “root level”, “effectiveness”, “treatment”
	Safety	“risks”, “unintended consequences”, “change child”, “side effects”, “working then stopping”
Feelings	Support	“hopeful”, “prayers”, “interesting”, “positive”, “exciting”, “worth a try”, “optimistic”, “yes”, “grateful”, “curious”
	Caution	“uncertain”, “concern”, “unsure”, “unknown", “ethics”, “frightening”, “distrust”, “nervous”, “sad”, “newness”

^*^illustrative words/phrases are all direct quotes from participants

### Theme 1: Quality of life

Caregivers described what impact gene therapy could have on both the caregiver and their dependant.

#### 1a Symptoms

Caregivers described some of the difficulty their dependents face in everyday life and how gene therapy could perhaps alleviate some of the symptoms their dependant struggles with, perhaps help their dependant to avoid difficulties they experience. Caregivers also wondered how their dependant with FXS may adapt to any changes they experienced themselves after receiving gene therapy.

“I want to live in a world where everyone is valued however I see my son struggle on a daily basis with his anxiety, sensory needs, communication and other issues associated with fragile X so the thought of him being free from all this would be amazing”

“could bring relief to some of the more limiting issues that are part of my sons diagnosis”

“desperate for something that could profoundly help ease all my daughters anxiety and behavioural issues”

“they deserve to have a chance of alleviating their anxieties, behaviours and general lack of understanding of the world and the people in it. To maybe see them improve slightly would be amazing for them”

“I don’t know how he would react to feeling ‘different’”

“we would be worried that it might make him unhappy with his life and his limitations”

#### 1b Future

Many of the caregiver responses reported that they felt gene therapy could improve both their and their dependant’s life, perhaps make their life easier and lead to a ‘normal life’.

“if gene therapy could help release him it can only be a good thing”

“we get glimpses of what he’d be like if his brain could develop normally. Research that increases the likelihood and degree of independence should be supported”

There were some concerns from a few that gene therapy may change their dependant’s personality.

“initially I was all for it before discussing it further in that it could drastically change our sons personality among other things”

“…how can we change one thing and not expect other aspects to be changed as well”

#### 1c Tolerability

Some hesitance was discussed around how gene therapy would be given and how their child would manage treatment procedures. Caregivers discussed causing their child anxiety or pain.

“method of delivery and the co operation required by the participant”

“..ability to communicate feelings/pain”.

“Nature and frequency of physical intervention e.g. level of pain or discomfort”

“travel to new location, exposure to unfamiliar individuals, exposure to unfamiliar places”

### Theme 2: Outcomes

Caregivers described what the result of gene therapy may be and the potential scope of it may be.

#### 2a Cure

The concept of ‘cure’ was mentioned often with the idea of cure being positive. Caregivers described addressing the root cause, helping to reduce caregiver burden and providing their dependant with a better future.

“any pathway to treatments or cure should be explored”

“...I have waited a lifetime for progress in treatment and a cure”

“only promising way for a cure”.

“excited to see where that pathway takes us and if it can be the first real cure to the underlying cause”

#### 2b Safety

Unknown side effects were mentioned repeatedly and the unknown risks involved with gene therapy. It was mentioned that there could perhaps be unintended consequences and that it may work for a period of time and then stop working.

“taking part would depend on the risk involved and whether it would cause discomfort or pain”

“safety and efficacy would be our top concerns. There would have to be prior studies and trials completed showing proven benefits”

“I would want him to have it ASAP as long as its safe”

“high risk of irreversible consequences”.

### Theme 3: Feelings

Gene therapy in a very emotive subject for caregivers with many feelings used to describe their opinions. The majority providing lots of positive feelings towards gene therapy taking place and used words such as excited, interested and hopeful. Some responses were broadly supportive however used mixed emotions such as wanting gene therapy to take place however feeling cautious about it or had some trepidation.

“positive, then guilt/fear as if thinking I could change them makes me a bad person”

“I feel hopeful and excited about the possibilities gene therapy can unlock. At the same time conscious of unintended and profound consequences”

“curiosity about the possibilities, mixed with trepidation about the unknown”

#### 3a Support

Words like hopeful, positive, exciting, worth a try, optimistic were used repeatedly throughout the questionnaire. There were also words like interesting and curious.

“sounds exciting and promising”.

“I pray for this everyday”.

“ I have high hope gene therapy will be available soon”

“I feel hopeful for the future of my son when I hear about gene therapy”

#### 3b Caution

A few people reported negative feelings at the idea of gene therapy and distrust for it as a treatment option, feelings of being frightened or unsure were reported alongside feeling nervous and uncertain.

“Not sure, cautious, don’t want anything to harm my child”

“I don’t trust gene therapy at all”

“Sad, I do not agree with it”

## Discussion

In this study, we aimed to elicit what caregivers’ views on gene therapy and overall we found that respondents felt interested, excited and hopeful about gene therapy, with the majority saying they want research into gene therapy for FXS to take place and that they would consider it as a treatment option. It is also important to note that, while many gave positive views on gene therapy, some responses mixed this with caution and there were also a small number of responses who did not want gene therapy to be explored as a treatment option. It should also be noted that the results come from a group who self-reported a wide range of understanding of genetic therapies; it is not clear how this may affect the findings.

The hopefulness of gene therapy potentially being a ‘cure’ was raised by many of the respondents which echoes research into other genetic neurodevelopmental conditions, such as Rett syndrome [17]. Respondents felt that gene therapy could help to provide a better future for their dependant and provide them with a degree of independence in their future. There were repeated mentions of hope that gene therapy could provide help with anxiety and behavioural issues– features often mentioned in the literature and frequently described as being distressing or difficult [7, 9]. They also felt that it would reduce what some describe as the caregiver burden, an issue which is discussed frequently in other literature [1, 3, 17, 19]. Caregivers also highlight the current lack of treatment options available to people with fragile X syndrome and the idea that gene therapy provides hope to the FXS community that something may be able to help with the daily struggles that they face. Previous research has highlighted that the priorities for families for effective treatments include anxiety, learning and behaviours [21]

Potential negative effects of gene therapy raised included immediate side effects such as pain or discomfort and acute safety issues, as well as more long-term unintended consequences, such as change to their personality– how much’FXS’ affects their personality and would gene therapy remove part of ‘them’. Similarly, how would the affected individual react to potentially feeling different; perhaps having increased awareness of their differences could in fact result in them being ‘less well’, despite what may be regarded as an improvement in symptoms or improvement in cognition. Could this perhaps lead to them being aware of social stigma resulting in lowered self-esteem or anxiety and depression? This is a concern that is somewhat touched upon in the literature [12], however the long-term emotional and psychological implications of increasing awareness in individuals with intellectual disabilities have not been fully explored. Given the potential for such outcomes, future studies should investigate the delicate balance between cognitive improvements and the associated challenges to provide a more comprehensive understanding of the risks and benefits. Concern about side effects is also highlighted as a concern in a similar study carried out in Rett syndrome where respondents also specifically raised the same concern that their child would change and whether this is something that their dependant would want [17]. Such questions will be difficult to answer until such time as trials of gene therapy in FXS have actually started. It is important to note that concern around side effects is not exclusive to gene therapy. Previous research has shown that concern about potential side effects, including similar immediate and longer-term negative effects, is one of the most frequently reported barriers to taking part in clinical trials in FXS, [2, 5, 6, 18].

It was notable that respondents had very mixed levels of knowledge about gene therapy and what it might actually entail. Although the potential positive and negative outcomes require further research to elicit, should clinical trials be planned, it would be important to provide more information to communities around what these might be. Information around the mechanisms of gene therapy and the practicalities of vector delivery, including the likely invasive nature, would also be important to provide to allow communities to discuss developments from an informed standpoint. Related to this, although not directly raised by participants in the current study, the issue of affected individuals being unable to give informed consent has been previously brought up in regard to participating in clinical trials more broadly [6] so it is also likely to be an important consideration moving forward.

A number of potential limitations to the current work should be considered. Respondent bias is probably the largest potential confounder of this type of research and we do not have data around the background of the respondents so we are unable to state they are representative of the fragile X community as a whole. Similarly, although, this questionnaire was circulated through a variety of routes, including the UK family support organisation for FXS, it is possible that respondents are those most interested in hearing about novel therapies. It is also possible that those with strong negative opinions did not answer the questionnaire as they are not favour of such research. We did not calculate inter-coder reliability due to the inductive nature of our thematic analysis and it is possible that other researchers would identify different themes. Although our findings indicate a broad positivity towards research into genetic therapies for FXS, it is possible that this may be affected by participant characteristics, such as age, sex or phenotypic differences. Future research should examine whether there are subgroups within the community with different views around such therapies. It is also important to note that, at present, there are no approved gene therapies for neurodevelopmental disorders and it is likely that community views will evolve over time as more information about possible outcomes and side effects become available. Finally, although this study sheds some light on how caregivers feel about gene therapy, there remains no information available from those people who are actually affected directly by FXS. Future research should also explore their views about taking part in clinical trials of gene therapy, or indeed of other therapies.

## Conclusion

Overall, this study showed a strong interest from caregivers of those with FXS in gene therapy trials taking place and optimism about the impact it could have on their family. Caregivers feel hopeful, excited and interested in the prospect of gene therapy potentially providing a new treatment option. There is also some trepidation about unintended consequences, the newness of the treatment and tolerability. Information programmes targeted at caregivers would be helpful to allow communities to have informed discussions around gene therapy. These should cover the different types of genetic therapies, the practicalities (e.g. modes and frequency) of vector delivery, and the potential benefits and risks of therapies. Should future trials of gene therapy for FXS become a reality, then consideration of these caregiver views, both positive and negative, will help to shape how trials are delivered in a way that best meets the needs of the fragile X community.

## Supplementary Information

Supplementary Material 1.

## Data Availability

No datasets were generated or analysed during the current study.
