# Supplementary material for: Preliminary perspectives on gene therapy in fragile X syndrome: a caregiver view
Source: J Neurodev Disord. 2025 Sep 1;17:55. doi: 10.1186/s11689-025-09629-1 (PMC12400708; doi:10.1186/s11689-025-09629-1)
Supplement: Supplementary file 1 — Supplementary Material 1. [file 11689_2025_9629_MOESM1_ESM.docx]

We are keen to hear from you what your thoughts are on gene therapy and its potential use in Fragile X Syndrome (FXS).

While various interventions to help people with FXS are available, there are currently no medicines or other therapies that target the underlying genetic change which causes the condition. Gene therapy represents a different therapeutic approach as it specifically targets the underlying genetic factors which lead to FXS.

Early studies in laboratories have shown that gene therapy might be helpful in FXS, though at present no research has been done in people. However, there are human studies of gene therapy being done in other genetic neurodevelopmental conditions (e.g. Rett syndrome) raising the possibility that gene therapy for FXS might become a reality in the future.

We are aware that for some people this could be a controversial subject, so we think it is important that families affected by FXS get the chance to give their views about gene therapy. We are therefore grateful to you for answering the following questionnaire.

You can find out more about gene therapy by using the following link

<https://www.esgct.eu/gene-therapy-basics>

**ABOUT THE PERSON YOU CARE FOR:**

Firstly, we would like to ask some questions about the person with FXS who you care for.

**Location?**

Country of residence

**What is the age of the person with FXS who you care for?**

0 – 5

6 – 10

11 – 15

16 – 20

20 >

**Is the person you care for:**

Male

Female

**Below are some behaviours that can be associated with FXS. Please rate them between 0 and 10 on how these affect the person you care for day to day.**

0 = not a problem

10 = high (has a profound effect on day to day life)

Aggression

Anxiety

Attentional difficulties

Hyperactivity

Language/communication

Learning

Repetitive behaviour

Self injurious behaviour

Sensory sensitivities

Social behaviour

**Has the person you care for previously been involved in a clinical trial?**

Yes

No

**ABOUT YOU**

Next, we would like to ask some questions about you.

**Are you?**

A parent of someone with a diagnosis of FXS?

A carer of someone with FXS

**YOUR VIEWS ON GENE THERAPY**

**What is your initial reaction when you hear about gene therapy?**

**How would you describe your understanding of gene therapy?**

**Do you think that research into gene therapy for FXS should occur? Please give reasons for your answer**

**What factors would influence you when considering whether or not to suggest the person you care for takes part in a clinical trial involving gene therapy?**

**Would you consider gene therapy as a treatment option for the person you care for? Please give reasons for your answer.**

**What concerns, if any, do you have about gene therapy?**

**Please use the space below if there is anything else you would like to share about your thoughts on gene therapy in Fragile X Syndrome**
